# The Role of the Baldwin Effect in the Evolution of Human Musicality

**DOI:** 10.3389/fnins.2017.00542

**Published:** 2017-10-06

**Authors:** Piotr Podlipniak

**Affiliations:** Institute of Musicology, Adam Mickiewicz University in Poznań, Poznań, Poland

**Keywords:** Baldwin effect, human musicality, cortico-subcortical loops, pitch structure, musical rhythm

## Abstract

From the biological perspective human musicality is the term referred to as a set of abilities which enable the recognition and production of music. Since music is a complex phenomenon which consists of features that represent different stages of the evolution of human auditory abilities, the question concerning the evolutionary origin of music must focus mainly on music specific properties and their possible biological function or functions. What usually differentiates music from other forms of human sound expressions is a syntactically organized structure based on pitch classes and rhythmic units measured in reference to musical pulse. This structure is an auditory (not acoustical) phenomenon, meaning that it is a human-specific interpretation of sounds achieved thanks to certain characteristics of the nervous system. There is historical and cross-cultural diversity of this structure which indicates that learning is an important part of the development of human musicality. However, the fact that there is no culture without music, the syntax of which is implicitly learned and easily recognizable, suggests that human musicality may be an adaptive phenomenon. If the use of syntactically organized structure as a communicative phenomenon were adaptive it would be only in circumstances in which this structure is recognizable by more than one individual. Therefore, there is a problem to explain the adaptive value of an ability to recognize a syntactically organized structure that appeared accidentally as the result of mutation or recombination in an environment without a syntactically organized structure. The possible solution could be explained by the Baldwin effect in which a culturally invented trait is transformed into an instinctive trait by the means of natural selection. It is proposed that in the beginning musical structure was invented and learned thanks to neural plasticity. Because structurally organized music appeared adaptive (phenotypic adaptation) e.g., as a tool of social consolidation, our predecessors started to spend a lot of time and energy on music. In such circumstances, accidentally one individual was born with the genetically controlled development of new neural circuitry which allowed him or her to learn music faster and with less energy use.

## Introduction

Human musicality can be understood as a set of abilities which enable people to recognize and produce music (Fitch, 2015). Although the worldwide diversity of music reveals the cultural flexibility of *Homo sapiens* in musical behavior, the fact that people in all known cultures sing (Nettl, 2000) and can recognize music without explicit learning (Tillmann, 2005) suggests that musicality is a part of human biological endowment (Blacking, 1973; Fitch, 2006, 2015). In fact, a variety of theories have been proposed which try to explain the possible adaptive value of music (Cross and Morley, 2008). Many of the possible biological values of music have been put forward such as increasing sexual attractiveness (Darwin, 1871; Miller G. F., 2000), facilitating mother-infant bonds (Dissanayake, 2008), enhancing group consolidation (Roederer, 1984; Storr, 1992; Harvey, 2017), reducing cognitive dissonance (Perlovsky, 2010, 2012), and alerting outsiders about a group cohesiveness (Hagen and Bryant, 2003; Hagen and Hammerstein, 2009), to name only the most popular ideas. However, music is a complex communicative phenomenon (Zimmermann et al., 2013) which is composed of many features. Some of these features are shared with other communicative phenomena, which raises the question about music specificity and in consequence, about the biological character of human musicality. For example, the manipulation of sound intensity, stress and tempo is an important part of all human songs but also of speech. Moreover, a similar use of these features is observed among the vocal expressions of many mammalian species (Zimmermann et al., 2013) including our closest relative—the chimpanzee (*Pan troglodytes*) (Slocombe et al., 2009). The patterns of continuously modulated sounds used as a tool of expression and induction of emotions in other individuals are called “expressive dynamics” (Merker, 2003) or “affective prosody” (Zimmermann et al., 2013) and seem to be evolutionarily more ancient than any species of hominins. Of course, music is also usually composed of such elements which are not shared with other forms of human sound expressions. The interpretation of musical stimuli in terms of pitch classes and temporal isochrony are at least two musical elements which are absent in speech (Fitch, 2013a) and other human vocalizations. Although pitch and the duration of vowels can serve as discreet phonological units in tonal and time sensitive languages respectively (Remijsen and Gilley, 2008; Wong et al., 2012; Remijsen, 2014) both pitch and vowel length in speech lack hierarchical ordering based on mental reference points. Such a hierarchy is evident in music where pitch classes are organized in reference to pitch center (Podlipniak, 2016) and rhythm measures in reference to musical pulse (London, 2012). By taking into account that both of these features are not present in the vocal expressions of chimpanzees it is reasonable to assume that they were also absent in the vocal repertoire of the common ancestor of chimpanzees and humans and thus are relatively evolutionarily young innovations.

The coexistence of different evolutionarily aged features in our musical expression encourages us to think of human musicality in an analogous way, as in the faculty of language (Hauser et al., 2002) namely in terms of musicality in the broad and narrow sense. While musicality in the broad sense can encompass a set of abilities which develop at an early stage of human ontogenesis, and which allow the identification of pitch contour, changes in tempo, and dynamics as parts of the affective prosody (Zimmermann et al., 2013), only abilities which constitute musicality in the narrow sense enable the recognition of musical structure. From this perspective the actual question about the origin of music is related to the origin of these abilities which constitute musicality in the narrow sense. What makes music a phenomenon distinguishable from other experiences of sounds is the interpretation of sounds by the human nervous system not just as unrelated sound events but as a psychologically unified musical structures. Although it has been proposed that musical structure (e.g., melody) was discovered by early humans due to the resemblance of musical sounds to the acoustic characteristics of human vocalizations (Purves, 2017), the real problem is why the nervous system of early humans started to interpret certain acoustic parameters as discrete pitch and rhythm units organized into syntactically based sequences. After all, despite the fact that a sound stimulus is usually a very complex phenomenon composed of an enormous number of spectral and temporal cues, different species recognize their species-specific vocalizations using only the subsets of features which are distinctive solely to their song cognition (Bregman et al., 2016; Shannon, 2016). In other words, every species is sensitive to specific acoustic cues due to the proclivities of its own nervous system. In addition, speech and music, two complex hierarchical cognitive systems of *H. sapiens* (Fitch, 2014), operate with a restricted number of different distinctive features (Patel, 2008). Therefore, there must be something which predisposes humans to focus their attention on particular acoustic features whilst ignoring others. What is more, the vast majority of musical structures, especially songs of tribal communities (Blacking, 1973), are organized according to certain syntactic rules (Koelsch, 2013 but see London, 2011), which suggests that musical syntax is a natural trait of human vocalization. Apart from this, musical syntax based on pitch classes and rhythmic units measured in reference to musical pulse is a music-specific feature (Fitch, 2013a). This raises the question about the evolution of the abilities which allow the recognition of musical syntax, which seems to be the core of human musicality.

## Musical syntax as a music specific feature

The human ability to organize and interpret stimuli as syntactically complex sequences is often regarded as a milestone in the evolution of human cognition (Hauser et al., 2002; Fitch, 2014). Even though syntax, understood in the broad sense as rules combining discrete elements into sequences (Patel, 2008), can be attributed to certain animal songs (Okanoya, 2013; Suzuki et al., 2016), both musical and language syntaxes seem to be exceptional in terms of their complexity and function (Fitch and Jarvis, 2013). Musical syntax however, is often thought of as a derivative of language syntax (Patel, 2008 but see Jackendoff and Lerdahl, 2006), or as a product of domain-general structural computation (Fitch, 2014; Van de Cavey and Hartsuiker, 2016) rather than a functionally separate phenomenon. In fact, there are good reasons to assume the general role of structural computation in the processing of language and musical syntaxes. There are many neuroimaging studies that show an overlap in the activation of cortical structures (located in the inferior frontal gyrus) during the processing of musical and language syntaxes (Patel et al., 1998; Maess et al., 2001). Moreover, there is also research which reveals that the same structures (e.g., Broca's area) play a certain role in the performing of other tasks involving sequential ordering (Tettamanti and Weniger, 2006; Friedrich and Friederici, 2009; Higuchi et al., 2009; Wakita, 2014). Also, the cognitive deficits observed after lesions in the Broca's area include not only deficits in production and recognition of language syntax but also action execution and observation as well as musical syntax processing (Fadiga et al., 2009).

However, from a behavioral point of view, language and music are functionally different phenomena. The former communicates referential meaning by the means of intersubjectively understandable concepts (Bickerton, 2010), whereas the latter exchanges information which is at least more ambiguous in terms of its semantic content (Cross, 2005). Since natural selection operates on phenotypes, we can assume that syntactical language evolved because it gave an advantage to our ancestors over those individuals who could not grasp the syntactic rules present in the language of our ancestors. This means that in the case of cognitive abilities natural selection acts directly upon the behavioral effects of the activity of brain structures. Therefore, when searching for the evolutionary origin of a particular cognitive trait it seems more reasonable to look at it from Tinbergen's perspective (Tinbergen, 1963), namely by considering the possible ultimate function of the brain products (e.g., syntactical language), rather than the proximal function which a particular brain structure fulfills in the processing of a certain task or tasks. From this point of view, the circuitries which perform whole task such as the processing of syntactical language should be assumed as the result of natural selection rather than isolated brain structures which take part in the operations of these circuitries (e.g., Broca's area). Since evolution usually optimizes organisms by the means of adjusting the existing traits to new functions (Jacob, 1977), it is not surprising that the same brain structures are often parts of functionally different circuits. Of course, the human brain is characterized by having huge plasticity, thus the general ability to attribute “tree structures” (Fitch, 2014) (complex syntax) to stimuli, is not restricted solely to language and music. It is well known that people are able to implicitly learn the artificial syntaxes of different stimuli (Reber et al., 1999). Nonetheless, in comparison to artificial grammars, both language and music seem to be exceptional in respect to the rate and easiness of the implicit learning of their syntactic rules by children (Jablonka and Lamb, 2005; Tillmann, 2005). The fact that there is no culture without language and music additionally strengthens the point of view that musicality, similar to language abilities, is a natural part of human behavior rather than being a very old cultural invention similar to writing or playing chess.

### Musical syntax vs. language syntax

There are many similarities between language and musical syntaxes. Both are compositional and hierarchical (Merker, 2002) and both generate long-distance dependencies (Bickerton, 2009; Woolhouse et al., 2016). The default mode of language—speech—is like music in the auditory domain, and it has been observed that the processing of music and speech syntactic tasks activates the peri-Sylvian network which connects the inferior frontal gyrus with sensory cortices located in the temporal lobes (Fitch, 2014). However, musical and language syntaxes are also quite different in many respects. First of all, music is composed of different units to those of language. The basic units of speech are phonemes which are experienced in our internal world as unique qualities hardly comparable with our experience of pitch class. Their discrimination is also based on different spectral cues. Pitch classes are recognized by the fundamental frequency of harmonic sound (F_0_) (Stainsby and Cross, 2008) whereas phonemes mainly by the spectral and temporal shape of sound (Xu et al., 2005). Although the processing of certain characteristics of spectral shape is important for the discrimination of timbre in music (McAdams and Giordano, 2008), the role of timbre in musical syntax is at least doubtful. Admittedly, timbre can play an important role in the structural organization of music as it is observed in certain musical styles such as in the deep throat singing of Tuva and Mongolia (Levin and Süzükei, 2006), the music of the Jew's harp (Fox, 1988), and tabla music in India (Patel, 2008). There are also musical cultures (e.g., Yakut culture in Siberia) in which the structure-forming function of pitch is extremely reduced whereas timbre seems to be a dominant factor which structures the sound order. Nevertheless, in all these cases timbral structure is hardly comparable to pitch and rhythm structure mainly because of the multidimensional perceptive character of timbre (Lerdahl, 1987). Also, our mental images of timbre in music and phonemes in speech differ, although both are based on the interpretation of the spectral shape of sound. Therefore, even though language grammar (Lerdahl and Jackendoff, 1983), prosodic structure (Heffner and Slevc, 2015) as well as phonotactics and morphonotactics are to some extent comparable to musical syntax (Lerdahl, 2013), the perceptual salience of these phenomena is disparate. The crucial difference between language and musical syntax however is related to the function which these syntaxes fulfill in music and language being different communicative phenomena. In language, syntax is mapped into conceptual meaning (propositional semantics; Hilliard and White, 2009) which allows a concatenation of meaning i.e., putting together two or more units and thereby creating a new meaning in comparison to the meanings of those units alone (Bickerton, 2009). But this process of mapping does not seem to be unidirectional as semantics can influence syntactic rules as in the case of some verbs in which the meaning determines grammatical patterns (Dor and Jablonka, 2000). Thus, the function of language syntax is strictly related to communication of specific conceptual meanings (Dor and Jablonka, 2001). In contrast, the function of syntax in music has nothing to do with such a complex interdependence between syntax and concepts observed in language. Although there is an endless dispute over the existence and character of musical semantics (Patel, 2008; Koelsch, 2013; Reybrouck, 2013; Seifert et al., 2013) even if one admits that music can communicate referential meaning, both the type of this meaning (Dor and Jablonka, 2001) and its relation to musical syntax (Lerdahl, 2013) are definitely different than in language.

### Musical rhythm and pitch as the basis for musical syntax

There is currently no agreement about what musical syntax actually is (Patel, 2003; London, 2011; Koelsch, 2013; Lerdahl, 2013; Asano and Boeckx, 2015; Heffner and Slevc, 2015). The majority of research on musical syntax has been conducted on Western artistic music—especially on the functional relations between chords (Koelsch, 2013). But the case of Western artistic music seems to be an inadequate example of human musical expressions as the manifestation of *H. sapiens* musicality (Jackendoff and Lerdahl, 2006). After all, functional harmony is an exception within the wide variety of world music. Although music based on functional harmony has become more and more widespread in the last century, its history is very young in comparison to the ancient history of music without functional harmony. Nevertheless, the experience of even simple melody without any accompaniment necessitates the recognition of syntactic relations. These relations are hierarchical, meaning that the sequence of sounds is interpreted by the nervous system as being composed of units (sounds perceived as belonging to a particular pitch class and having a particular rhythmic measure) which possess different prominence. This prominence attributed to sounds as elements of metrical (London, 2012; Fitch, 2013b) and pitch (Lerdahl and Jackendoff, 1983; Huron, 2006) patterns is a mental construct as with the prominence of grammatical categories in language. However, in the case of music the metrical or tonal prominence is rather “felt” than “conceptually known” as in language cognition. This preconceptual character of musical hierarchy is especially evident when music is experienced by non-musicians who, in contrast to professional musicians (Burns and Ward, 1978), do not always recognize pitch intervals by the means of categorical perception (Smith et al., 1994). However, even musically trained listeners experience musical hierarchy in such a preconceptual way despite the fact that they are additionally able to recognize musical structure in terms of precise mental categories. What seems to be a source of the preconceptual experience of musical hierarchy is somehow related to motor and emotional brain processes. The recognition of meter in music can be understood as a kind of entrainment which exists in the connection between our auditory and sensorimotor systems (London, 2012). It has even been proposed that human metrical interpretation of music is based on hidden sensorimotor activity (Repp, 2007). This sensorimotor activity during listening to music leads in turns to emotional reactions (Sievers et al., 2013). Also, the recognition of pitch hierarchy causes measurable emotional reactions (Steinbeis et al., 2006; Koelsch et al., 2008; Mikutta et al., 2015), and perception of pitch changes (often described as “leaps” and “steps”) can lead to sensorimotor interpretation (Nikolsky, 2015). Since emotion is an evolutionarily old motivational mechanism (Toates, 1988), the function of which is to assess a potential danger or attractiveness of perceived stimuli (Panksepp, 1998), the tight connection between musical structure and emotions suggests the biological importance of this human specific interpretation of sound in terms of pitch and metric hierarchy.

### Musical syntax and emotions

During the processing of musical syntax, people experience a set of subtle emotional reactions (emotional qualia) dependent on the position of a note in each syntactic context (Huron, 2006; Margulis, 2014). This observation is supported by the fact that the bilateral Amygdalae and the orbitofrontal cortex are differently activated during listening to music depending on the syntactic relations of musical sequences (Mikutta et al., 2015). In spite of the coexistence of an affective experience during listening to musical syntax, the emotional reaction to musical syntax is often suggested as the mere result of cognitive recognition of syntactic structure (Koelsch et al., 2008) rather than an integral part of this recognition. For example, Huron (Huron, 2006) has proposed that the association of emotions with particular pitch classes is the result of the so called “misattribution effect.” In the case of music it is the misattribution of limbic reward or punishment (caused by fulfilling or not fulfilling predictions about which pitch class will be next) to the pitch classes themselves depending on the general mechanism of prediction. However, in the original misattribution effect (Dutton and Aron, 1974) the feeling experienced in response to a stimulus (e.g., the instability of a bridge perceived by the vestibular and visual systems) is misattributed to another stimulus (e.g., a woman perceived by sight). But in Huron's example there is only one stimulus—sound. Because prediction is the ultimate function of the nervous system (Llinás, 2001) one can assume that every perception is based on prediction. The limbic reward (or punishment) in response to well predicted (or falsely predicted) stimuli is an evolutionarily old mechanism of the assessment of stimuli (Panksepp, 1998), inseparable from every perception. In other words, both the emotional reaction to a particular stimulus and the prediction of stimuli are parts of cognition. Therefore, the prediction processes of the nervous system cannot be treated similarly to external stimuli as a source of emotions. The actual source of emotion is an external stimulus and a prediction process is one of the mechanisms, the function of which is to deliver information about the external world that is assessed by emotions. Moreover, if the emotional effect was solely the result of prediction then the emotional reactions to equally predicted stimuli should be the same, independent of whether they are parts of e.g., musical syntax, speech phonotactics or the sequence of timbres in music (Gorzelańczyk et al., 2017). Yet the emotional experience of musical syntactic relations seems qualitatively different from the experience of phonotactics and other syntactically organized sequences. This difference is evident if we compare singing with speech. Although both of these vocal expressions are composed of syntactically organized sounds, the variations of pitch in time occur much slower in singing than in speech (Zatorre and Baum, 2012) and the emotional impact of singing on listeners seems to be greater in comparison with speech from our childhood (Nakata and Trehub, 2004) and lasting throughout our lives.

Taking into account the behavioral specificity of singing, both prediction and emotional reactions to successive sounds are in this case rather the integral parts of a mental tool dedicated to processing musical structure. From this perspective, different subtle emotional reactions to a variety of possible syntactic relations are the elements of a functionally specific form of communication, similar to how semantics is strictly connected with grammar in natural language. In other words, music can be understood as a mapping system in which syntactical relations are mapped into preconceptual emotional cues. For the majority of the human population, the recognition of musical syntax is a solely preconceptual experience. Of course, the conceptual level of musical syntax recognition is achievable by professional musicians. However, in contrast to implicitly learned tacit syntactic knowledge by non-musicians, this additional level of competence necessitates strenuous explicit learning. Changes in the neural architecture of musicians in response to environmental influences (explicit learning) could perhaps represent a kind of phenotypic adaptation within the cognitive domain which is possible due to the brain's plasticity (Moreno and Bidelman, 2014; Strait and Kraus, 2014). In fact, it has been observed that music performance affects the transcription of genes that are related to dopaminergic neurotransmission, motor behavior, neuronal plasticity, and neurocognitive functions such as learning and memory (Kanduri et al., 2015) which can be responsible for the observed differences between musicians and non-musicians. Therefore, the fact that musical syntax can be recognized by musically trained individuals at a conceptual level shows how much cultural influence can extend cognitive skills rather than saying something about its primordial nature. Since for average humans the syntactic relations in music, both metrical prominence (especially evident when they dance or tap), and pitch prominence (when they sing), are somehow felt but are difficult to express conceptually, it is reasonable to assume that motor, emotional and cognitive processing are an integral part of the ability to recognize and produce syntactically organized music.

## Music and the baldwin effect

The main problem concerning the evolution of the abilities to use syntax as a part of any communicative system is related to the question about the cause of “syntax genes” proliferation (Dor and Jablonka, 2001). If musical or language syntax is adaptive due to extending communicative capabilities, then this means that it must be used by at least two individuals. After all, as long as one individual cannot use syntax in communication with another individual, the use of syntax by the latter is useless. Even with the use of music in the form of self-communication, as in the case of “personal song” in the musical cultures of Siberia, Far East, and Amerindian tribes (Nikolsky, 2015), the development of the abilities to organize music syntactically necessitates learning from another individual. However, the appearance of a new genetic trait in the population is usually a result of accidental mutation or recombination. Because the probability of the coincidence of identical mutations of the same allele in the same generation is very low the appearance of a genetically based predisposition to organize vocal expression syntactically seems puzzling. In other words, all advantages of syntax are useless in a population in which only one individual is able to produce and recognize syntactically organized sequences. A possible solution to this problem can be the Baldwin effect (Baldwin, 1896a; Simpson, 1953).

### The baldwin effect

The Baldwin effect is an evolutionary mechanism which transforms a culturally invented and acquired trait into an instinctive trait by the means of natural selection (Baldwin, 1896a; Simpson, 1953; Hall, 2001). Although this mechanism was independently proposed at the end of the Nineteenth Century by at least three different people (Baldwin, 1896a,b; Morgan, 1896; Osborn, 1896) it was forgotten with some exceptions (Simpson, 1953; Waddington, 1953a,b), for more than half of the next century. Only in the last few decades of the twentieth century has the Baldwinian idea started to inspire scientists and philosophers and has regained popularity (Godfrey-Smith, 2003). The core of this concept is very simple. Some animals due to their cognitive flexibility learn new adaptive behaviors in response to environmental changes. If a particular behavior is adaptive, lasts many generations, and its learning is strenuous and time-consuming, sooner or later a genetically based predisposition appears and starts to be favored by natural selection (Dor and Jablonka, 2000, 2010; Godfrey-Smith, 2003; Jablonka and Lamb, 2005). Therefore, the Baldwin effect is a combination of learning and the genetic assimilation of a learned trait (Dor and Jablonka, 2000, 2001; Godfrey-Smith, 2003). The process of Baldwinian evolution occurs in three stages: (i) the appearance of a new environmental challenge, (ii) the invention of a new behavior as a response to the new environmental challenge and its proliferation by the means of learning—at this stage natural selection favors cognitive plasticity, (iii) the appearance of a new genetically based predisposition (canalization, Jablonka and Lamb, 1995; Dor and Jablonka, 2010)—at this stage natural selection favors less flexible individuals but faster at exhibiting a particular adaptive behavior (Godfrey-Smith, 2003).

Since an important factor in the Baldwinian evolution of human behavior is the social character of our species, the aforesaid environmental challenge can be a part of the socio-cultural products of hominins. This specific socio-cultural environment often described as a “cultural niche” (Godfrey-Smith, 2003) has been proposed as a crucial element in the evolution of natural language (Bickerton, 2010; Deacon, 2010). In some regards, the evolution of language can represent a niche construction (Deacon, 1997, 2003)—a kind of niche extension or elaboration. The importance of this “cultural niche” seems evident when taking into account that at least during the last 2 million years hominins have become more and more socially complex animals in comparison to other primates (Dunbar, 2014). Living in a complex social group definitely causes new challenges which influence both natural selection and niche construction. In the process of niche construction hominin brains and hominin culture can be considered as a specific environment in which language evolved (Deacon, 2003). From this perspective the Baldwinian process is a part of the gene-culture coevolution (Lumsden and Wilson, 1982; Richerson et al., 2010; Gintis, 2011). Since speech (the “default mode” of language) is similar to music in many respects (both are transmitted by the acoustic domain, both are syntactical systems composed of discrete elements etc.), and language is assumed to be a crucial factor in the cultural evolution of *H. sapiens*, it seems reasonable to assume that the evolution of human musicality is somehow related to the evolution of language. However, language is a very elaborate signal related to the exchange of conceptual meaning. The presence of non-symbolic and non-conceptual culture in many other species (Cantor and Whitehead, 2013; van de Waal et al., 2013; Fehér et al., 2016) indicates that the beginning of cultural niche construction can be based on the exchange of preconceptual meaning. Therefore, music as an example of a communication system operating on preconceptual meaning is a good candidate to be a part of more ancient communicative tool other than language and so the proposed Baldwinian scenarios of language origin must differ from the possible Baldwinian processes that led to the emergence of music.

### The baldwinian evolution of music

Human musicality seems to be a very good example of the potential effects of Baldwinian evolution (Podlipniak, 2015). Huron has suggested that apart from certain reflexes, the human auditory experience is mainly influenced by learning (Huron, 2006). The predominance of learning in shaping human auditory cognition implies, according to Huron, that the auditory environment of hominins must have been very semiotically unstable. Following Huron's reasoning, such a semiotic instability led to the great variety of music found around the world. However, music as a product of human musicality is characterized not only by culture-specific features but also by universals (Nettl, 2000; Bispham, 2009; Brown and Jordania, 2011; Savage et al., 2015) which suggests that apart from the cultural (environmental) influence, the music-specific genetic constraints also shape the musical mind of every human. This means that on the one hand, musicality develops spontaneously and effortlessly but on the other hand, the learning of more sophisticated musical skills, as in the case of professional musicians, is time-consuming and necessitates a lot of effort. The transmission of musical information has its roots in the human ability of vocal learning which is exceptional among primates (Janik and Slater, 1997; Fitch and Jarvis, 2013). However, human vocal learning is canalized into an imitation of selected acoustic characteristics rather than the literal copying of every heard sound (Jackendoff and Lerdahl, 2006). People are very skillful at imitating the distinctive features of phonemes, the temporal order of sound sequences, and fundamental frequency of harmonic sounds but not very talented when they try to simulate the barking of a dog or environmental sounds such as the noise of a refrigerator which seem a very simple task for many parrots. This canalization suggests that apart from the aforementioned environmental instability, certain circumstances related to hominin vocal expressions must have been stabilized long enough during numerous generations to cause natural selection to have promoted an instinct to learn only selected sound features (Briscoe, 2000; Gibson and Tallerman, 2011). As a result, speech and music, similar to many songbirds' songs, are examples of so called ritual culture (Merker, 2009). The most important characteristic of ritual culture is its transmission by the means of imitative social learning (Merker, 2005, 2012). In contrast to non-imitative social learning, learning by imitation consists of copying the behavior of other individuals (Jablonka and Lamb, 2005). Therefore, what is important in the transmission of ritual is not the result of a particular action but the action itself (Merker, 2009). In the case of music, transmitted units are pitch classes and rhythm measures (Merker, 2002, 2003). After all, a melody is recognized independent of whether it is played slower or faster on the flute, piano, or when sung. What is important for the recognition of melodic pattern is its pitch and rhythm structure, not timbre or dynamics. In this respect music seems to be an even more striking example of ritual culture than speech in which, apart from poetry, what is crucial is the transmission of the semantic content of utterance and not its literal form. However, the thing that makes melody easier to remember is musical syntax, the evolution of which is most easily explained by the Baldwin effect.

The Baldwin effect may promote a particular trait due to different adaptive functions. For example, Morgan proposed that organic evolution (the term which Morgan used to describe the process known today as the Baldwin effect) can explain the origin of bird songs which evolved as a result of sexual selection (Morgan, 1891, 1920). Since bird songs are similar to human music in many respects, it is tempting to explain the origin of musical syntax by the means of the Baldwin effect in which the adaptive function of music is to attract sexual partners. If this is true, musicality could have been used by hominins as a mating handicap (a mark of the quality of a mate, Zahavi, 1975; Zahavi and Zahavi, 1997; Miller G. F., 2000) since the production and recognition of musical syntax is costly in terms of energy (necessary to process the perceived sounds and to control the vocal production of songs) and time spent on singing (which for example can be used for foraging instead). In such a scenario the syntactical complexity of a hominin song should attract females more than a song that lacks such a complexity due to the costliness of the song's complex structure being an indicator of fitness (Miller G., 2000). If these female preferences had been stable enough throughout many generations, the Baldwinian mechanism should have transformed the learning of culturally invented rules of musical structure into an instinct to learn and organize musical sounds in a syntactic way. This emerged instinct to learn the distribution of music-specific discrete elements based on intuitive recognition of their probability of occurrence would have left space for idiosyncratic song modifications similar to those observed in songbirds' behavior. Such a leeway in creating new songs allows the sustaining of the process of sexual selection based on the songs' complexity. However, a study of female preferences toward musical complexity showed that women do not have a tendency to prefer more complex music during and around ovulation (Charlton et al., 2012), which does not support the Baldwinian scenario of music origin based on sexual selection. Similarly, research shows that musical aptitude and achievements are not a predictor of mating success (Mosing et al., 2014). Of course these studies are not conclusive and more studies are necessary to test a possible role of sexual selection in music evolution. Nevertheless, so far the Baldwinian scenario in the sexual selection of human musicality needs more empirical support to be convincing.

Another possible scenario of the Baldwinian origin of music is related to the idea that music can serve as a tool of social consolidation. This scenario started the moment a new social challenge first appeared. The increasing size of the hominin population caused an increase in inter-individual and inter-group competition for food and other resources (Dunbar, 2014). One way to cope with this problem was to form alliances between individuals belonging to a group. This strategy has been observed in other primates, including our closest relatives—the chimpanzee (Mitani, 2009; Gilby et al., 2013), which suggests that hominins could use a similar strategy. Dunbar has proposed that as group size increased, grooming as the main tool to sustain social alliances became insufficient. Instead, hominin vocalizations started to serve as a tool of social consolidation (Dunbar, 1996). While this idea seems to be unconvincing as far as the origin of language is concerned (Dunbar and Lehmann, 2013; Grueter et al., 2013) its validity as an explanation of the origin of music still remains an open question. An increasing number of studies suggest that communal singing can facilitate social bonds (Dunbar et al., 2012; Tarr et al., 2014; Pearce et al., 2015, 2016, 2017), which supports Dunbars' idea. However, the precise mechanism of how music could act in this way remains a puzzle. Although the obtained results of the aforementioned studies must not necessarily be the effects of the adaptive value of social bonding, being for example a byproduct of sexually selected behavior, certain characteristics of musical syntax seem to bespeak the hypothesis of music as a tool of social consolidation. A big problem which afflicts individuals living in groups is the situation in which some individuals use resources obtained by other individuals—the so called “free-riding problem.” Communal song rituals demand that all participants must know the musical structure of the song. The learning of a particular song's structure is time-consuming and necessitates strenuous imitation of the vocal behavior of others especially in the case when hominins did not possess an instinct to learn musical syntax. Therefore, by devoting equal effort in order to learn a ritualized song, communal singing can serve as a good test of being prone to act together with others. After all, poor singers can be easily recognized which can lead to ostracism. In other words, the consolidation effect observed after communal singing can be a product of the unconscious assessment of other individuals in terms of their proclivity to being free-riders. From this perspective, a lack of synchronization hinders consolidation which can be a result of detecting potential free-riders and can lead to looking for new allies.

Also a proximal explanation of the mechanism responsible for the consolidating power of music can be related to observed characteristics of music processing by the human nervous system. It is possible that music consolidates individuals by the means of temporal and spectral synchronization between the brain states of co-performers (Bharucha et al., 2011). If this is true, our predecessors had to simultaneously imitate their vocalizations in order to sustain social trust (Podlipniak, 2016). This collective imitation became the beginning of a consolidating vocal ritual. Without any predisposition which canalized vocal learning so that hominins would have been sensitive to certain acoustic features, the process of the learning of vocal rituals would have been very strenuous and time-consuming. During this time, the second stage of Baldwinian evolution began in which natural selection preferred individuals who were characterized by the most flexible learning. In order to learn new melodies and sing them together then the appropriate predictions of what (which particular pitch class) and when (the position of a particular rhythm measure in relation to musical pulse) would happen in the near future was necessary. Syntax is exactly what makes the successful predictions of sound events during singing easier and, as a consequence, facilitates collective singing. At this stage, the learning of simple syntactic rules would have been accessible to hominins in a similar way to people that learn artificial syntaxes today. Because the costs of ritual learning were high an individual who was accidentally endowed with proclivities to predict the melody better than others gained an advantage over the rest of a group. In the long run, the progeny of this individual has dominated the whole population.

In a similar vein the Baldwin effect could have contributed to the origin of music if its adaptive function advertised the defending skills of a group (Hagen and Bryant, 2003; Hagen and Hammerstein, 2009; Jordania, 2014). However, in case an acoustic aposematism (instrumental music, singing) had been directed against predators (Jordania, 2014) a possible role of the Baldwinian mechanism in the origins of human musicality would have been restricted solely to the canalization of the elements of musical display which are recognizable by predators. These elements are a part of musicality in a broad sense such as pitch contour, changes in tempo, and dynamics rather than the syntactic relations specific to the discrete structure of human music. In the Baldwinian scenario, the initially invented complexity of musical syntax which became a part of the hominin cultural niche was most probably accessible (comprehensible) only to the hominin species. Therefore, predator reactions did not depend on the subtleties of musical structure and had not been a selective factor which could have influenced the process of canalization of musical-syntactical abilities which actually define musicality in a narrow sense. In contrast, if music was a coalition signaling display directed toward conspecifics (Hagen and Bryant, 2003), the Baldwinian scenario could have been very similar to that presented above in the case of consolidation as the adaptive function of music. The only difference is that this time the selective pressure which would have been responsible for the canalization of human musicality had been induced by the reactions of enemies. However, while rhythm syntax seems to contribute to the signaling/deteriorating function of music (despite the fact that people from different cultures can recognize different units of musical pulse in the same piece of music (London, 2012), a well synchronized rhythm is perceivable independent of whether the rules of rhythm syntax are familiar to us or not), the origin of pitch syntax is more problematic as a result of its possible adaptive signaling/deteriorating function.

First of all, even today when people are most probably endowed with an instinct to learn pitch system and pitch syntax, well spectrally synchronized music which is based on an unfamiliar pitch system can be perceived as being out-of-tune (Ellis, 1885), which can be a sign of a poor performance rather than a signal of a performers' coalition or consolidation. Additionally, the recognition of pitch syntax by contemporary humans depends on tacit knowledge about the statistical distribution of pitch classes in a particular music (Tillmann et al., 2000; Tillmann, 2005; Huron, 2006). Foreigners who listen to unfamiliar music usually experience different tonal qualia than people familiar with this music (Castellano et al., 1984; Kessler et al., 1984; Stevens, 2004, 2012; Curtis and Bharucha, 2009). Although this situation looks similar to the aforesaid difference in musical pulse perception, it differs in one important aspect. Pitch syntax is based on pitch hierarchy in which the most prominent place is pitch center, the experience of which is accompanied by the emotional qualia of completeness, resolution etc. There is nothing resembling pitch center in rhythm hierarchy. The misrecognition of actual tonal relations in reference to pitch center by foreigners can lead to divergence between the observed emotional expression of performers (also by the means of expressive dynamics) and tonal qualia felt by those foreigners. Such a divergence can also be a signal of poor performance. Importantly, without the canalized strategy to learn pitch syntax the differences in musical dialects between hominin groups would have been even greater than between modern geographically distant musical cultures causing the aforesaid divergence to be even greater. Therefore, while in the scenario of music origin in which music is a coalition signaling system the Baldwinian mechanism can explain the origin of musical rhythm so it is difficult to imagine a similar role of the Baldwin effect in the origin of pitch syntax as a result of its coalition signaling function.

It is worth mentioning however, that the possible different adaptive functions of music are not mutually exclusive and the Baldwin effect could have played an important role at different stages of the gradual process of the evolution of human musicality. Nevertheless, it seems that Baldwinian evolution was necessary at least in the process that led to the emergence of the complex musical syntax as a part of hominins' singing behavior.

## The evolution of new circuitry

The appearance of ritualized singing behavior among hominins required the development of new abilities and recruitment of existing skills. An important ability which had to be a necessary condition of the development of culturally variable vocal communication is the aforesaid vocal learning (Janik and Slater, 1997). This ability had to be present at the first stage of the Baldwinian evolution of human musicality. The similarity of vocal pathways in vocal learning birds to cortical–basal ganglia–thalamic–cortical loops in humans suggests the role of the latter in the processing of speech (Jarvis, 2007). In fact, there is an increasing number of studies which emphasize the role of the basal ganglia in the processing of language (Booth et al., 2005) especially in the learning of language during childhood (Krishnan et al., 2016). The fact that language impairment can be the result of neurodevelopmental deficits of the corticostriatal loops (Krishnan et al., 2016) shows that corticostriatal connectivity might have been an important element of evolutionary change leading to the evolution of vocal communication among our predecessors. It is not surprising that the basal ganglia contributes to the processing of sound sequences specific to vocal communication. It is known that the basal ganglia is important in reinforcement learning (Bar-Gad et al., 2003) which is necessary to acquire the majority of culturally transmitted information. This ability is strictly connected to predictive functions which are related to internal timing (Dreher and Grafman, 2002). It is reasonable to assume that in the beginning hominin vocal communication was composed of simple vocal expressions which must have been vocally learned.

In the process of vocal learning the recognition and prediction of distinctive acoustic features is necessary and so the specific connections between the basal ganglia and auditory cortices had to be favored by natural selection during the second stage of the Baldwinian evolution of human musicality. Because contemporary humans are characterized by the ability to sustain and reproduce F_0_ which is crucial for singing (Bannan, 2012) but not for speaking, the sequencing of sounds based on their F_0_ characteristics must have become an important trait of hominin sound rituals. The consolidating function of them did not necessitate any referential conceptual meaning. Instead simple emotional cues were enough to establish close social relations. The emotional reinforcement of a well predicted sound i.e., the sound which is perceived as possessing a particular pitch and which happens at an exactly predicted point of time, started to act as a cue for social acceptance. The positive emotional reaction in response to music is in fact an evolutionarily old clue of social acceptance. It has been observed that this emotional reward occurring in response to music is related to the corticostriatal interactions involving auditory cortices and the nucleus accumbens (Salimpoor et al., 2013). Also the perception of beat in music is based on corticostriatal interactions (Grahn and Rowe, 2009, 2013). It is suspected that the role of the cortico-basal ganglia circuits in speech evolution is related to the positive selection of the FOXP2 gene variant (Enard, 2011). However, because the mutation of FOXP2 also impairs rhythm processing in music leaving intact pitch processing (Alcock et al., 2000; Tan et al., 2014) the evolution of the abilities to recognize pitch syntax must have been related to other genetic factors. Independent of what particular genetic factors influence pitch and rhythm processing in music (Tan et al., 2014), human perceptive preferences to recognize pitch classes and rhythm measures as parts of syntactically organized sequences suggest that at the last stage of the Baldwinian scenarios natural selection started to prefer individuals endowed with these canalized perceptive proclivities.

## Conclusion

The proposed possible Baldwinian scenarios of the evolution of human musicality solve the problem of the specificity of musical syntax which is functionally and structurally different from language syntax. This specificity suggests that all theories which explain the syntactic characteristic of music as a byproduct seem unconvincing. After all, music seems to be the only spontaneously emerging syntactic system apart from speech, dance (Opacic et al., 2009), and some drum (Winter, 2014) and whistled languages (Carreiras et al., 2005; Güntürkün et al., 2015; Meyer and Busnel, 2015). Although the details of these proposed scenarios are speculative, future research can elucidate which particular elements of these scenarios are more probable. The most promising would be studies which concentrate on the comparison between the functions of cortico-striatal loops (Gorzelańczyk, 2011) in speech and music processing. In order to investigate which particular loop is mostly involved in the processing of musical syntax, neuroimaging studies could be conducted in which the activity of limbic and dorsolateral-prefrontal loops can be compared during performing tasks related to the recognition of musical and language syntaxes. Although the complex interactions between genetic, epigenetic, and cultural information which occur in evolution have not so far been explained in detail (Jablonka and Lamb, 2005) it seems important to consider them in future models of the evolution of human musicality. The rapidly advancing development of genomics and transcriptomics allows us to expect that the details of these complex interactions will be better understood in the near future.

Another possible study which could be conducted to test the proposed Baldwinian origin of musicality is to compare the results of singing syntactically simple with syntactically complex (more demanding in terms of explicit learning) tonal melodies. If singing syntactically complex melodies leads to a greater consolidation of singers or if the singing group is assessed by others as being more consolidated than singers of the syntactically simple melodies then it would suggest that the tendency which was proposed as the main source of the Baldwinian evolution of musicality is still present in the human population. Also the comparison of singing tonal melodies by a group of people with other collective sound expressions such as simultaneously reading prose, reciting poetry, drumming, and singing atonal melodies in free rhythm (without musical syntax) would be informative as far as the question of what particular features of human sound expressions are responsible for the observed effects. If the proposed consolidating function of human musicality is tenable, then the consolidating effect of singing tonal melodies and drumming should be greater than other collective behaviors. Nevertheless, in order to better understand the origin of music the broad holistic view of human musicality is necessary. This view should be based on integrated knowledge taken from such disciplines as genetics, evolutionary biology, paleoanthropology, neuroscience, psychology, archeology, ethnomusicology and cognitive musicology.

## Author contributions

The author confirms being the sole contributor of this work and approved it for publication.

### Conflict of interest statement

The author declares that the research was conducted in the absence of any commercial or financial relationships that could be construed as a potential conflict of interest.
